# Differences in sequences between HBV-relaxed circular DNA and covalently closed circular DNA

**DOI:** 10.1038/emi.2017.41

**Published:** 2017-06-21

**Authors:** Magda Rybicka, Anna Woziwodzka, Tomasz Romanowski, Piotr Stalke, Marcin Dręczewski, Krzysztof Piotr Bielawski

**Affiliations:** 1 Department of Molecular Diagnostics, Intercollegiate Faculty of Biotechnology, University of Gdansk and Medical University of Gdansk, Abrahama 58, Gdansk 80-307, Poland; 2 Department of Infectious Diseases, Medical University of Gdansk, Smoluchowskiego 18, Gdansk 80-214, Poland

**Keywords:** cccDNA, HBV, MALDI-TOF MS, polymorphism, RCDNA

## Abstract

The hepatitis B virus (HBV) genome exists in two forms: circular covalently closed DNA (cccDNA) and relaxed circular DNA (RCDNA). Here, we investigated the presence of differences in the sequences of both forms in paired samples of serum and liver tissue. The serum and liver biopsy samples were collected at the same time from 67 chronically infected patients. The genotyping of the RCDNA and cccDNA was performed using mass spectrometry analysis. The HBV mutations located in the HBV pol (P) and the HBV basal core promoter/pre-core (BCP/PC) regions were included. The BCP/PC and P sequences of the RCDNA extracted from liver and blood samples were different in 39% and 16% of patients, respectively. Differences were also found between RCDNA and cccDNA extracted from the same liver specimen. Moreover, the cccDNA BCP/PC region sequence had an impact on various virological and clinical parameters. We demonstrated that there are differences between the RCDNA and cccDNA sequences that were extracted from the same liver tissue. However, further investigations are needed to analyze whether the mutations in the cccDNA are conserved and whether cccDNA serves as a ‘mutation storage’ pool for HBV. This result could have profound implications for the subsequent therapy choices for treatment-experienced patients.

## INTRODUCTION

Infection by the hepatitis B virus (HBV) affects approximately two billion people worldwide and is still a global health problem. In total, 240–350 million chronic HBV carriers could develop chronic hepatitis B (CHB) with a high risk of liver cirrhosis and hepatocellular carcinoma (HCC)—the second leading cause of cancer death worldwide. It has been estimated that patients with CHB are at a 100-fold higher risk for developing HCC compared to healthy individuals. Despite the availability of an effective vaccine that can be used as a primary preventive strategy against this virus, hepatitis B remains an essential health problem, causing ~780 000 deaths per year.^1, 2, 3^

The main challenge in the management of CHB patients is the lack of efficient treatment strategies that could achieve HBV elimination. This lack reflects the insufficient knowledge of the covalently closed circular DNA (cccDNA) persistence reservoir of HBV. The main goal of antiviral therapy is the prevention of liver disease progression and, eventually, the prolonged survival of patients. Current treatments are limited to two major classes of antiviral agents: pegylated interferon-alpha (PEG IFNα) and five nucleos(t)ide analogs (NAs) including lamivudine (LAM), telbivudine (LdT), entecavir (ETV), adefovir (ADV) and tenofovir (TDF or TAF).^2, 4, 5, 6^ Despite the finite duration of treatment and the higher probability to achieve sustained virological response (defined as a loss of HBsAg) with PEG IFNα, its use is limited by its high cost and numerous side effects. In addition, a significant proportion of patients do not respond to this treatment. Moreover, numerous immunocompromised and end-stage liver disease subjects have contradictions to PEG IFNα therapy. NAs are safe, generally well tolerated and can suppress viral replication to undetectable levels. This treatment may reduce the risk of disease progression, and can result in liver fibrosis and cirrhosis regression. However, a long-term treatment, which is required to maintain virological control, often leads to the selection of drug-resistant mutants that harbor amino acid substitutions in the viral polymerase or provoke serious side effects (nephrotoxicity, osteopenia, bone marrow aplasia, etc.).^2, 7, 8^ Moreover, the HBV population in infected individuals consists of a diverse pool of genetically heterologous variants, known as quasispecies. A viral population changes over time under selective pressure, which includes the host immune response and/or the presence of antiviral drugs. The most viable HBV strain with the highest replication capacity in the environment is likely to become the dominant variant. However, the dominant strain could be previously absent in the quasispecies population.^7, 9^

Recently, there have been numerous studies regarding the relationship between the presence of HBV mutant variants and the analog therapy response. To date, there is no clear evidence that the baseline HBV heterogeneity influences the treatment efficacy.^4^ However, in our previous studies, we demonstrated that it is not the presence but the amount of the resistant variant, in comparison to the wild-type (WT) virus, that is crucial for the antiviral strategy success.^10, 11^

In this study, we sought to compare the polymorphisms in two HBV DNA forms, cccDNA and relaxed circular DNA (RCDNA), in serum and liver biopsy samples of CHB patients.

## MATERIALS AND METHODS

### Patients and clinical samples

This study enrolled 67 consecutive patients with CHB that were referred to the Department of Infectious Diseases, Medical University of Gdansk and Hepatology Outpatients Clinic, Pomeranian Centre for Infectious Diseases and Tuberculosis in Gdansk in 2014–2016 (Table 1). The study group consisted of treatment-naive patients (57/67) and treatment-experienced patients (10/67). The treatment-experienced patients were between 20 and 87 years of age. The majority of subjects (80%, 8/10) had received PEG IFNα in the past, and 20% (2/10) of patients were previously treated with LAM. The therapy effect was assessed after 48 weeks of PEG IFNα administration, and the LAM treatment duration ranged from 48 to 52 weeks, in accordance with the National Health Fund (NFZ) recommendation. The local Medical Ethics Committee approved the study protocol, and all patients provided written informed consent to participate. The liver biopsies and blood samples were collected from each patient at the same time. Whole blood samples were collected into Vacutainer tubes without anticoagulant and incubated for 45 min at room temperature. After clotting, the samples were centrifuged at 3500*g* for 15 min, and the resulting supernatant (serum) was transferred into a fresh Eppendorf tube. Next, the serum was aliquoted into cryovials and stored at −20 °C until further analysis.

The liver specimens were divided into two parts: one part was used for routine histopathological examination, and the other was stored at −80 °C until use. The liver specimens that were dedicated to histological examination were preserved in 10% buffered formalin and routinely transferred to a paraffin block. Several stains including hematoxylin and eosin, Masson’s trichrome for collagen, Gomori’s stain for reticulin and Prussian blue for iron were done. Two independent pathologists, experienced in hepatopathology, assessed the inflammation activity and stages of fibrosis, iron deposits and steatosis according to Scheuer score.^12^

### Sample preparation

The viral DNA was extracted from 200 μL of serum samples using a High Pure Viral Nucleic Acid Kit (Roche Diagnostics, Penzberg, Germany), as described before.^10^ To extract the cccDNA from the serum, a QIAamp DNA Mini Kit (Qiagen, Hilden, Germany) was used. The following modifications were introduced to the manufacturer’s protocol: the incubation time was extended to 3 h, and 1 mg/mL of poly(A) (Roche Diagnostics) was used as a carrier. The HBV DNA (both forms) from liver biopsy samples was extracted with a High Pure PCR Template Preparation Kit (Roche Diagnostics) with a slightly modified protocol: the incubation time with Proteinase K was 18 h instead of 10 min, 1 mg/mL of poly(A) was used to increase the viral DNA yield and the final elution volume was 40–80 μL (depending on the biopsy sample weight).

The genotyping of the RCDNA form was done directly after DNA extraction. For the cccDNA analysis, two different procedures were applied. At first, the cccDNA was amplified by rolling circle amplification (RCA) with the same conditions as described by Margeridon *et al.*,^13^ followed by its digestion with the *Spe*I enzyme (New England Biolabs, Ipswich, MA, USA). After the separation of the digestion products on a 1% agarose gel, all bands corresponding to the cccDNA were purified and analyzed by the mass spectrometry.

In the second procedure, HBV DNA was treated with *T5* exonuclease (Epicentre, Madison, WI, USA), which degrades linear or circular dsDNA in the 5′ to 3′ direction. This enzyme does not degrade supercoiled dsDNA but has a ssDNA endonuclease activity. After this digestion step, the samples were purified using a QIAquick PCR Purification Kit (Qiagen). Positive (pAM6 plasmid, ATCC, Manassas, VA, USA) and negative (HBV DNA External Quality Control—EQC, PeliSpy PRO; AcroMetrix, Thermo Fisher Scientific, Waltham, MA, USA) controls were used at each step. The pAM6 plasmid contains the full genome of the HBV subtype adw, genotype A. The EQC is intended for use with assays that are designed to detect the HBV DNA in human plasma from donations of whole blood and blood components for transfusion.

### Genotyping

The PCR and specific-primer single base extension (SBE) primers for each investigated HBV variation were designed using the Assay Design Suite v2.0 (Agena Bioscience, San Diego, CA, USA) as previously described.^10, 14^ All oligonucleotides were ordered unmodified, with standard purification.

Next, mass spectrometry analysis was performed to compare the RCDNA and cccDNA sequences. HBV mutations located in the HBV pol (P) region that are associated with drug resistance and mutations located in the HBV basal core promoter/pre-core region (BCP/PC) were included (Table 2).

The detection of mutations was carried out with the MassARRAY system following standard protocols (Agena Bioscience) with minor modifications. The 25 μL (instead of 5 μL) PCR reaction mixture contained: 200 nmol of each primer, 5 μL of HBV DNA, 1 × HotStar Taq buffer, 1 mmol/L additional Mg^2+^, 0.2 mM dNTP and 1 U of HotStar *Taq*DNA Polymerase (Qiagen). The PCR conditions were similar to those described previously except that the extension time was shortened to 30 s. After amplification, all PCR products were cleaned with shrimp alkaline phosphatase.^10, 14^ Next, four extension reactions (12-plex in assay 1, 17-plex in assays 2 and 3, and 14-plex in assay 4) using iPLEX Pro chemistry were conducted according to standard procedure (Agena Bioscience). All extension products were treated with a resin to remove contaminants and dispensed onto a 96-spot SpectroChip (Agena Bioscience). HBV WT and mutant variants were then discriminated via matrix-assisted laser desorption/ionization time-of-flight mass spectrometry (MALDI-TOF MS). All data analyses were performed with the Typer Analyzer Application, version 4 (Agena Bioscience).

### Statistical analysis

The statistical analysis was performed using STATISTICA data analysis software, version 12.0 (StatSoft, Tulsa, OK, USA). All statistical data are presented as a mean±standard error of means (±SE) or as a median value. The standard error was used because the distributions of data were skewed. The relationships between variables were estimated using the Spearman's rank correlation coefficient, Mann–Whitney’s *U* test, univariate logistic regression and multiple regression. When the *P*value was <0.05, the result was considered statistically significant.

## RESULTS

### The presence of HBV cccDNA

With the use of RCA, which imitates the natural replication strategy of circular DNA molecules, HBV cccDNA was detected in 1/67 serum and 48/67 liver biopsy samples. The HBV serological markers, viral load and hematological variables were not correlated with the presence of the cccDNA form.

**Figure 1:**
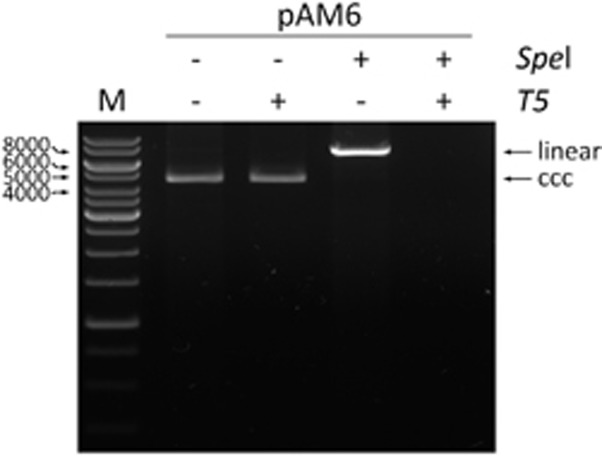
The evaluation of the *T5* exonuclease digestion. In each case, 1 μg of the pAM6 plasmid (7.5 kb) was used. The linearization of the plasmid was performed with 10 units of *Spe*I enzyme (NEB, Ipswich, MA, USA) at 37 °C for 1 h. The *T5* exonuclease digestion was performed with 10 units of the enzyme (Epicentre) at 37 °C for 30 min. The positions of the linear and covalently closed circular (ccc) plasmid forms are indicated. The electrophoresis was carried out in 0.8% agarose gel in 1 × TAE buffer. M indicates the DNA marker.

In the second procedure, the relaxed circular HBV DNA was removed by *T5* exonuclease. We used a purified plasmid DNA construct, pAM6 (ATCC), that contained a full-length HBV monomer that served as the standard for optimizing the digestion of the RCDNA (Figure 1). This plasmid had been previously calibrated against the World Health Organization HBV standard (plasmid HBV-A) and showed the same amplification efficiency.^15^ Finally, 10 μL of the HBV DNA that was extracted from liver and 25 μL of the HBV DNA that was extracted from serum were treated with 10 units of *T5* exonuclease. With this optimized procedure, the cccDNA form was shown to be present in all 67 liver biopsy samples and in none of the serum samples.

Considering that the cccDNA should be present in all liver samples from CHB patients, we analyzed the RCDNA and cccDNA sequences after digesting the samples with *T5* exonuclease. The RCA procedure was regarded as not reliable in our study.

### HBV heterogeneity

Background variant calling was verified with pAM6 plasmid at a broad range of concentrations, both alone and in a mixture with genomic DNA. The obtained results were fully concordant with the reference nucleotide sequence of the plasmid, and the presence of genomic DNA did not influence the variant calling. Within the analyzed serum samples, from 0.02 to 478 000 kIU/mL viremia were detected. All samples were easily analyzed by MALDI-TOF MS. The HBV mutants were found in 88% of treatment-naive and 80% of treatment-experienced patients. HBV pol (P) region and HBV basal core promoter/pre-core region mutants (BCP/PC) in treatment-naive patients were detected at sites nt379, nt400, nt512, nt616, nt700, nt750, nt766, nt772, nt814, nt841, nt886, nt895, nt1613, nt1762, nt1764, nt1858, nt1896 and nt1899. The most common HBV variants were A1899G (44%), G1764A (40%), A1762T (38%), G1896A (26%) and C1858T (25%). In the group of experienced patients, the HBV mutants were also detected at sites nt367, nt373 and nt846. The most common mutants were A1762T (62%), G1764A (62%), A400C (50%), G616A (50%), G886A (50%), C1858T (38%) and G1896A (38%). No mutant variants were found at sites nt700 and nt1613 for experienced patients.

The presence of the C1858 WT variant in patients’ sera was strongly associated with a higher liver fibrosis stage (C vs. T: sex-adjusted odd ratios (OR)=0.045, 95% confidence interval (Cl)=0.004–0.50, *P*=0.009) as well as with an advanced liver inflammation level (C vs. T: sex-adjusted OR=0.055, 95% Cl=0.005–0.61, *P*=0.015). Moreover, the serum HBV G1613A variant was associated with a higher viral load (*R*=0.342, *R*^2^=0.117, adjusted *R*^2^=0.100, *P*=0.0098).

### Differences in HBV RCDNA and cccDNA sequences

**Figure 2:**
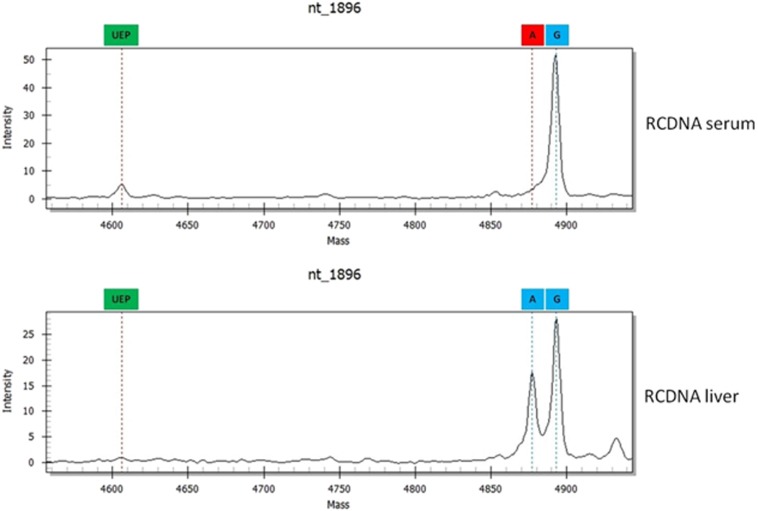
Differences between HBV RCDNA forms extracted from liver and serum samples. Abbreviations: circular covalently closed DNA, cccDNA; hepatitis B virus, HBV; relaxed circular DNA, RCDNA.

The BCP/PC and P sequences of the RCDNA extracted from liver and blood samples were different in 39% (26/67) and 16% (11/67) of patients, respectively (Figure 2). These patients had significantly higher HBV DNA levels (sex-adjusted OR=4.22, 95% Cl=1.18–15.09, *P*=0.024). In total, 54 differences were found, and they involved mutations at nt1764 (23%), nt1762 (17%), nt1899 (15%), nt1896 (11%), nt886 (10%), nt1858 (6%), nt700 (4%), nt1613 (2%), nt373 (2%), nt379 (2%), nt616 (2%), nt741 (2%), nt814 (2%) and nt841 (2%). All differences were confirmed by direct sequencing (Supplementary Figure S1 and Supplementary Table S1). The HBV deviations from the RCDNA sequences in WT mixtures that were extracted from liver specimens were most frequently found at nt1764 and nt1762, whereas in serum RCDNA, the variations among the HBV quasispecies were most common at nt1899 and nt1896 (Table 3).

**Figure 3:**
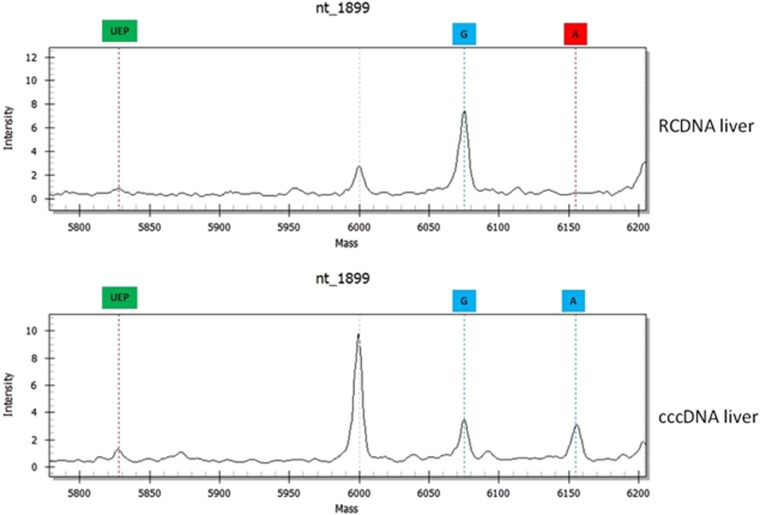
Differences between HBV RCDNA and cccDNA extracted from the same liver specimen. Abbreviations: circular covalently closed DNA, cccDNA; hepatitis B virus, HBV; relaxed circular DNA, RCDNA.

Differences were also found between RCDNA and cccDNA extracted from the same liver specimen. Forty-three percent (29/67) of these samples differed in the BCP/PC region and 28% (19/67) in the pol region (Figure 3). In total, 61 differences were found, and they involved mutations at nt1764 (16%), nt1762 (16%), nt1899 (15%), nt886 (11%), nt1858 (10%), nt1896 (8%), nt895 (6%), nt400 (5%), nt750 (3%), nt373 (2%), nt616 (2%), nt700 (2%), nt741 (2%) and nt814 (2%).

HBV variations in WT mixtures only in the HBV RCDNA that was extracted from liver specimens were found at nt895 and nt1762; variations that occurred more frequently in the RCDNA than the cccDNA were found at nt1764 and nt1896. On the other hand, HBV quasispecies that were more common in the viral persistence reservoir (cccDNA) had mutations at sites nt400 (100%), nt1858 (80%), nt886 (67%) and nt1899 (55%) (Table 4).

The presence of the G1613 variant in the liver RCDNA sequence was significantly correlated with advanced liver fibrosis stages (G vs. A: sex-adjusted OR=0.08, 95% Cl=0.007–0.89, *P*=0.034) and higher HBV DNA levels (*R*=0.371, *R*^2^=0.137, adjusted *R*^2^=0.119, *P*=0.009) in the group of patients who had never previously been treated.

A statistical analysis revealed that the sequence of the cccDNA has an important influence on numerous virological and clinical parameters in treatment-naive patients. The existence of the G1764 HBV variant was associated with iron deposits in liver specimens (G vs. A: sex-adjusted OR=0.098, 95% Cl=0.02–0.70, *P*=0.017). The liver inflammation grade was dependent on possessing an HBV variation at nt1762 (A vs. T: sex-adjusted OR=0.18, 95% Cl=0.04–0.96, *P*=0.039), nt1858 (C vs. T: sex-adjusted OR=0.22, 95% Cl=0.07–0.70, *P*=0.012), nt1613 (G vs. A: sex-adjusted OR=0.023, 95% Cl=0.001–0.47, *P*=0.006) and nt1899 (G vs. A: sex-adjusted OR=0.06, 95% Cl=0.005–0.69, *P*=0.019). In addition, 1858T (C vs. T: sex-adjusted OR=0.12, 95% Cl=0.03–0.53, *P*=0.004), 1762T (A vs. T: sex-adjusted OR=0.14, 95% Cl=0.03–0.76, *P*=0.018), and 1764A (G vs. A: sex-adjusted OR=0.10, 95% Cl=0.02–0.63, *P*=0.012) variants were more frequently found in the cccDNA of patients with a higher liver fibrosis stage. Moreover, patients with the HBV 1899A variant had increased aspartate aminotransferase (ALT) levels (G vs. A: sex-adjusted OR=0.10, 95% Cl=0.013–0.79, *P*=0.025).

### Therapy response

To date, 19 patients started antiviral treatment: ten were administered PEG IFNα, seven ETV and two were treated with TDF. So far, none of these patients have finished antiviral therapy. Among patients treated with the nucleoside/nucleotide analogs (NAs), those receiving TDF monotherapy have achieved undetectable levels of HBV DNA and normalized serum ALT levels at 24 weeks of therapy. No resistance mutations were present in the RCDNA and cccDNA in these patients. Only one patient who was treated with ETV had detectable HBV DNA at 24 weeks of therapy. In this patient, HBV mutant variations were present in the RCDNA (liver and serum) and cccDNA at sites nt400, nt616, nt1613, nt1762, nt1764, nt1858 and nt1899. The HBV quasispecies were identified in both HBV DNA forms at nt1762, whereas a single variation was present only in the cccDNA (1762T). In the cccDNA, 400CA variations in the WT mixture were detected; in contrast, a single variation was observed in the RCDNA. All remaining patients that received ETV had an undetectable viral load at 24 weeks of treatment. No resistance mutations were detected in the cccDNA and RCDNA in these patients.

## DISCUSSION

Currently, effective therapies for CHB that quickly suppress HBV to undetectable levels in the bloodstream of most patients are available. Unfortunately, none of the drugs are potent enough to eliminate the viral reservoir in the hepatocytes. The loss of immune control of chronic persistent infection is responsible for treatment failure and most of the clinical complications of the disease. The major barrier to HBV eradication is the presence of the viral replicative form (cccDNA). HBV virons contain the RCDNA form of the genome, which is converted in the hepatocyte nuclei to fully double-stranded cccDNA after infection. During chronic HBV infections, the cccDNA accumulates in the hepatocyte nuclei (1–50 copies per cell), where it functions as the template for transcription, serving as a reservoir for future replication cycles of the virus. Thus, the RCDNA form is synthesized on the basis of the cccDNA template through the reverse transcription of a pregenomic RNA (pgRNA) intermediate. The nucleocapsids that enclose the progeny RCDNA are further assembled with envelope proteins and secreted outside the cell (as infective virons) or redirected to the nuclei to increase the cccDNA pool. Data in the literature clearly show that cccDNA is responsible for HBV reactivation following drug cessation and upon immunosuppression. Therefore, a lifelong treatment is required in most cases, and antiviral drug resistance represents a serious potential complication. The presence of resistant HBV mutations usually causes a virological breakthrough (any increase in serum HBV DNA) as well as a clinical breakthrough manifested by acute exacerbations of disease (ALT elevations), faster progression to liver failure and liver transplants, and a higher risk of HCC and death.^16, 17^

Another serious problem regarding HBV insensitivity to antiviral treatment is the viral memory of resistance. As more potent drugs with high barriers to resistance are available, a few studies have shown that if people have HBV that was resistant to one or more drugs in the past, such viral strains were still present but at relatively low proportions and could be detected only with highly sensitive methods.^11, 18^ The high-genetic barrier drugs are effective against the drug-resistant HBV variants, but economic conditions force the use of cheaper low-genetic barrier drugs in some countries.

The results from our analysis describe for the first time that RCDNA and cccDNA HBV forms may have different sequences. An important finding, that the variations between the RCDNA from the liver and blood samples that were extracted from the same patient were detected in 39% (BCP/PC) and 16% (P) of the study participants, has a clear explanation. Namely, the absence of mutations in plasma does not always exclude the possibility that clinically significant levels of HBV mutations are present at lower levels (<1%) within the hepatocytes of infected patients. These HBV drug-resistant variants that were not found in the blood have been removed and probably conserved deep within the hepatocytes, in the viral persistence reservoir—cccDNA. It should also be noted that drug-resistant strains have lower replication potential; however, BCP/PC mutations usually improve the replication capacity. In addition, identified differences between the sequences of the RCDNA and cccDNA extracted from the same liver specimen may support the hypothesis that the HBV cccDNA acts as a mutation storage pool for HBV. Another confirmation of our supposition is the significant importance of the cccDNA BCP/PC region sequence that, in this study, has an impact on various virological and clinical parameters (such as HBV viral load, liver inflammation grade and fibrosis stage, and the presence of HBeAg as well as the iron deposits in a liver biopsy) in the treatment-naive patients. In the case of the RCDNA, only one HBV mutant (G1613A) was associated with these particular parameters. As the HBV variations in the BCP/PC region of cccDNA are more important than those identified in the RCDNA, we suppose that the same situation occurs in the case of the P region. Taking into account those differences between the two HBV genomic forms in this region that were identified, we believe that the HBV mutations in cccDNA are more likely to be important for the antiviral therapy response. Moreover, we think that the mutations in the cccDNA form are highly conserved during the life cycle of HBV and they can quickly emerge in the blood when patients start low-genetic barrier antiviral treatment.

As none of the patients have finished antiviral treatment, at this moment it is impossible to analyze which HBV DNA form (cccDNA or RCDNA) is superior for determining the therapy response. Such information could have profound implications for the subsequent therapy choices for treatment-experienced patients.

## Figures and Tables

**Table 1:** Baseline demographic and clinical characteristics of patients

	**Study group (*****n*****=67)**
Age, years	32.44±15.74
Sex, % female	31%

*Liver grading*	
0.5	16%
1	47.5%
1.5	17%
2	16%
2.5	3.5%

*Liver staging*	
0.5	28%
1	39%
1.5	11%
2	14%
2.5	5%
3.5	3%
	
HBV DNA, kIU/mL^a^	12 847±8305
HBsAg, % positive	100%
HBeAg, % positive	6%
anti-HBe, % positive	71%
ALT, IU/L	31±9.48
AST, IU/L	25±12
GGT, IU/L	21±29

Abbreviations: alanine aminotransferase, ALT; aspartate aminotransferase, AST; gamma-glutamyl transferase, GGT.

a Mean value±standard error (SE).

Data are presented as a median value±interquartile range (IQR).

**Table 2:** HBV polymorphisms analyzed by MALDI-TOF MS

**HBV region**	**Amino acid substitutions**	**Nucleotide changes**
pol	rtT70S	A/T@337
	rtN71T	A/C@341
	rtS78T	T/A@361
	rtL80I(V)	C/T/A/G@367
	rtL82M	C/A@373
	rtV84M	G/A@379
	rtS85A	T/G@382
	rtA86P	G/C@385 A/C@400
	rtP92L	C/T@404
	rtT128N	C/A@512
	rtH133L	A/T@527
	rtS/T135C	A/T@532 C/G@533
	rtI/V163V	A/G@616
	rtF166L	T/C@ 625
	rtI169T	T/C@635
	rtV173L	G/C/T@646
	rtP177L	C/T@659
	rtL179P	T/C@665
	rtL180M	C/T/A@667
	rtA181T(V/S)	G/A/T@670 C/T@671
	rtT184G	A/G@679 C/G@680
	rtV191I	G/A@700
	rtA194T	G/A@709
	rtA200V	C/T@728
	rtS202I	G/T@734
	rtM204V/I	A/G@739 G/C/T@741
	rtM204S	T/G@740 G/T@741
	rtV207I	G/A@748 G/A/T/C@750
	rtS213T	T/A@766
	rtV214A	T/C@770
	rtQ215S	C/T@772
	rtS219T	T/A@784
	rtF221Y	T/A@791
	rtS223A	T/G@796
	rtI224V	A/G@799
	rtL229V/M	T/G/A@814
	rtI233V	A/G@826
	rtH234Q	T/A/G@831
	rtL235I	T/C/A@832
	rtN236T	A/C@836
	rtP237H	C/A@839
	rtN/S/H/A238S	A/C/G@841 T/C@843
	rtY245S	A/C@863
	rtN/H248H	A/C@871
	rtM250V	A/G@877
	rtV/I253I	G/A@886 A/G@895
Basal core promoter/pre-core		A1899G
		G1896A
		C1858T
		A1762T
		G1764A

**Table 3:** Differences identified in RCDNA sequence extracted from blood and liver samples of chronic hepatitis B patients

**Serum**	**WT**		**MIX**		**MUT**	
**Liver**	**MIX**	**MUT**	**WT**	**MUT**	**WT**	**MIX**
nt373	1	0	0	0	0	0
nt379	0	0	1	0	0	0
nt616	0	0	0	1	0	0
nt700	0	0	2	0	0	0
nt741	0	1	0	0	0	0
nt814	0	0	1	0	0	0
nt841	0	0	0	1	0	0
nt886	2	0	0	2	0	0
nt1613	0	0	1	0	0	0
nt1762	2	0	1	0	1	5
nt1764	1	2	2	0	2	5
nt1858	1	0	2	0	0	0
nt1896	1	0	3	0	2	1
nt1899	1	0	6	0	0	1

Abbreviations: HBV wild-type variant presence, WT; HBV mutant variant presence, MUT; wild-type and mutant HBV variants presence, MIX.

**Table 4:** Differences identified between RCDNA and cccDNA sequence extracted from liver samples

**RCDNA**	**WT**		**MIX**		**MUT**	
**cccDNA**	**MIX**	**MUT**	**WT**	**MUT**	**WT**	**MIX**
nt373	0	0	1	0	0	0
nt400	0	0	0	0	0	3
nt616	0	1	0	0	0	0
nt700	1	0	0	0	0	0
nt741	0	0	0	0	1	0
nt750	1	0	1	0	0	0
nt814	0	0	1	0	0	0
nt886	3	1	2	0	0	1
nt895	0	0	4	0	0	0
nt1762	0	3	1	5	1	0
nt1764	3	0	4	0	3	0
nt1858	2	1	1	0	0	2
nt1896	2	0	2	1	0	0
nt1899	5	0	4	0	0	0

Abbreviations: HBV wild-type variant presence, WT; HBV mutant variant presence, MUT; wild-type and mutant HBV variants presence, MIX.
