# Supplementary material for: Differences in sequences between HBV-relaxed circular DNA and covalently closed circular DNA
Source: Emerg Microbes Infect. 2017 Jun 21;6(6):e55–. doi: 10.1038/emi.2017.41 (PMC5520316; doi:10.1038/emi.2017.41)
Supplement: Supplementary Table S1 [file emi201741x1.doc]

| SAMPLE ID | HBV DNA form | nt_1613 | nt_1762 | nt_1764 | nt_1858 | nt_1896 | nt_1899 | nt_367 | nt_373 | nt_379 | nt_400 | nt_512 | nt_616 | nt_700 | nt_741 | nt_750 | nt_766 | nt_772 | nt_814 | nt_841 | nt_843 | nt_886 | nt_895 |
| --- | --- | --- | --- | --- | --- | --- | --- | --- | --- | --- | --- | --- | --- | --- | --- | --- | --- | --- | --- | --- | --- | --- | --- |
| 1 | seum RCDNA | G | A | G | C | G | G | T | C | G | A | C | G | G | G | G | T | C | T | A | C | A | A |
|  | liver RCDNA | G | A | G | C | G | G | T | C | G | A | C | G | G | G | G | T | C | T | A | C | A | A |
|  | liver cccDNA | G | A | G | C | G | G | T | C | G | A | C | G | G | G | G | T | C | T | A | C | A | A |
| 2 | seum RCDNA | GA | A | A | T | G | GA | T | C | G | C | C | A | G | G | G | T | C | T | A | C | A | G |
|  | liver RCDNA | GA | AT | A | T | G | GA | T | C | G | C | C | A | G | G | G | T | C | T | A | C | A | G |
|  | liver cccDNA | GA | T | A | T | G | GA | T | C | G | CA | C | A | G | G | G | T | C | T | A | C | A | G |
| 3 | seum RCDNA | G | A | G | C | G | G | T | C | G | A | C | G | G | G | G | T | C | T | A | C | A | A |
|  | liver RCDNA | G | A | G | C | G | G | T | C | G | A | C | G | G | G | G | T | C | T | A | C | A | A |
|  | liver cccDNA | G | A | G | C | G | G | T | C | G | A | C | G | G | G | G | T | C | T | A | C | A | A |
| 4 | seum RCDNA | G | A | G | C | G | G | T | C | G | A | C | G | G | G | G | T | C | T | A | C | A | A |
|  | liver RCDNA | G | A | G | C | G | G | T | C | G | A | C | G | G | G | G | T | C | T | A | C | A | A |
|  | liver cccDNA | G | A | G | C | G | G | T | C | G | A | C | G | G | G | G | T | C | T | A | C | A | A |
| 5 | seum RCDNA | G | A | G | T | A | G | T | C | G | A | C | G | G | G | G | T | C | T | A | C | A | A |
|  | liver RCDNA | G | A | G | T | A | G | T | C | G | A | C | G | G | G | G | T | C | T | A | C | A | A |
|  | liver cccDNA | G | A | G | T | A | G | T | C | G | A | C | G | G | G | G | T | C | T | A | C | A | A |
| 6 | seum RCDNA | G | A | G | C | G | G | T | C | G | A | C | G | G | G | G | T | C | T | A | C | A | A |
|  | liver RCDNA | G | A | G | C | G | G | T | C | G | A | C | G | G | G | G | T | C | T | A | C | A | A |
|  | liver cccDNA | G | AT | G | C | G | G | T | C | G | A | C | G | G | G | G | T | C | T | A | C | A | A |
| 7 | seum RCDNA | G | A | G | C | G | G | T | C | G | A | C | G | G | G | G | T | C | T | A | C | A | A |
|  | liver RCDNA | G | A | G | C | G | G | T | C | G | A | C | G | G | G | G | T | C | T | A | C | A | A |
|  | liver cccDNA | G | A | G | C | G | G | T | C | G | A | C | G | G | G | G | T | C | T | A | C | A | A |
| 8 | seum RCDNA | G | A | G | C | G | G | T | C | G | A | C | G | G | G | G | T | C | T | A | C | G | A |
|  | liver RCDNA | G | A | G | C | G | G | T | C | G | A | C | G | G | G | G | T | C | T | A | C | GA | A |
|  | liver cccDNA | G | A | G | C | G | G | T | C | G | A | C | G | G | G | G | T | C | T | A | C | GA | A |
| 9 | seum RCDNA | G | T | A | C | G | G | T | C | G | A | C | G | G | G | G | T | C | T | A | C | A | A |
|  | liver RCDNA | G | AT | GA | C | G | G | T | C | G | A | C | G | G | G | G | T | C | T | A | C | A | A |
|  | liver cccDNA | G | AT | GA | C | G | G | T | C | G | A | C | G | G | G | G | T | C | T | A | C | A | A |
| 10 | seum RCDNA | GA | A | G | C | G | G | T | C | G | A | C | G | G | G | G | T | C | T | A | C | A | A |
|  | liver RCDNA | GA | A | G | C | G | G | T | C | G | A | C | G | G | G | G | T | C | T | A | C | A | A |
|  | liver cccDNA | GA | A | G | C | G | G | T | C | G | A | C | G | G | G | G | T | C | T | A | C | A | A |
| 11 | seum RCDNA | G | A | G | T | G | G | T | C | G | C | C | A | G | G | G | T | C | T | A | T | G | A |
|  | liver RCDNA | G | A | G | T | G | G | T | C | G | C | C | A | G | G | G | T | C | T | A | T | G | A |
|  | liver cccDNA | G | A | G | CT | G | G | T | C | G | C | C | GA | G | G | G | T | C | T | A | T | GA | A |
| 12 | seum RCDNA | G | T | G | C | G | G | T | C | G | A | C | G | G | G | G | T | C | T | A | C | A | A |
|  | liver RCDNA | G | T | GA | C | G | G | T | C | G | A | C | G | G | G | G | T | C | T | A | C | A | A |
|  | liver cccDNA | G | T | GA | C | G | G | T | C | G | A | C | G | G | G | G | T | C | T | A | C | A | A |
| 13 | seum RCDNA | G | A | G | CT | G | G | T | C | G | A | C | G | G | G | G | T | C | T | A | C | A | A |
|  | liver RCDNA | G | A | G | CT | G | G | T | C | G | A | C | G | G | G | G | T | C | T | A | C | A | A |
|  | liver cccDNA | G | A | G | CT | AG | G | T | C | G | A | C | G | G | G | G | T | C | T | A | C | A | A |
| 14 | seum RCDNA | G | AT | GA | C | G | G | T | C | G | A | C | G | G | G | G | T | C | T | A | C | A | A |
|  | liver RCDNA | G | AT | GA | C | G | G | T | C | G | A | C | G | G | G | G | T | C | T | A | C | A | A |
|  | liver cccDNA | G | AT | G | C | G | GA | T | C | G | A | C | G | G | G | G | T | C | T | A | C | A | A |
| 15 | seum RCDNA | G | A | G | C | G | GA | T | C | G | A | C | G | G | G | G | T | C | TA | A | C | GA | A |
|  | liver RCDNA | G | A | G | C | G | GA | T | C | G | A | C | G | G | G | G | T | C | T | A | C | GA | A |
|  | liver cccDNA | G | A | G | C | G | G | T | C | G | A | C | G | G | G | G | T | C | T | A | C | A | A |
| 16 | seum RCDNA | G | A | G | CT | G | GA | T | C | G | A | C | GA | G | G | G | T | C | T | A | C | GA | A |
|  | liver RCDNA | G | A | G | C | G | G | T | C | G | A | C | G | G | G | G | T | C | T | A | C | GA | A |
|  | liver cccDNA | G | A | G | C | G | GA | T | C | G | A | C | G | G | G | G | T | C | T | A | C | GA | A |
| 17 | seum RCDNA | G | A | G | C | G | G | T | C | AG | A | C | G | GA | G | G | T | C | T | A | C | A | A |
|  | liver RCDNA | G | AT | A | C | G | G | T | C | G | A | C | G | G | G | G | T | C | T | A | C | A | A |
|  | liver cccDNA | G | T | G | C | G | G | T | C | G | A | C | G | GA | G | GT | T | C | T | A | C | A | A |
| 18 | seum RCDNA | A | T | A | T | G | A | T | C | G | A | A | G | G | G | G | T | C | T | A | C | A | A |
|  | liver RCDNA | A | T | A | T | G | A | T | C | G | A | A | G | G | G | G | T | C | T | A | C | A | A |
|  | liver cccDNA | A | T | A | T | G | A | T | C | G | A | A | G | G | G | G | T | C | T | A | C | A | A |
| 19 | seum RCDNA | G | A | G | C | G | G | T | C | G | A | C | G | G | G | G | T | C | T | A | C | A | A |
|  | liver RCDNA | G | A | G | C | G | G | T | C | G | A | C | G | G | G | G | T | C | T | A | C | A | A |
|  | liver cccDNA | G | A | G | C | G | GA | T | C | G | A | C | G | G | G | G | T | C | T | A | C | A | A |
| 20 | seum RCDNA | G | A | G | CT | AG | GA | T | C | G | C | C | G | G | G | G | T | C | T | A | C | G | A |
|  | liver RCDNA | G | A | G | CT | G | G | T | C | G | C | C | G | G | G | G | T | C | T | A | C | G | A |
|  | liver cccDNA | G | A | G | CT | G | G | T | C | G | C | C | G | G | G | G | T | C | T | A | C | G | A |
| 21 | seum RCDNA | G | A | GA | T | GA | GA | T | C | G | C | C | A | G | G | G | T | C | T | A | C | G | A |
|  | liver RCDNA | G | A | G | T | GA | GA | T | C | G | C | C | A | G | G | G | T | C | T | A | C | G | A |
|  | liver cccDNA | G | A | G | CT | GA | GA | T | C | G | C | C | A | G | G | G | T | C | T | A | C | G | A |
| 22 | seum RCDNA | G | T | A | C | G | G | T | C | G | A | C | G | G | G | G | T | C | T | A | C | G | A |
|  | liver RCDNA | G | TA | GA | C | G | G | T | C | G | A | C | G | G | G | G | T | C | T | A | C | G | A |
|  | liver cccDNA | G | T | A | C | G | G | T | C | G | A | C | G | G | G | G | T | C | T | A | C | G | A |
| 23 | seum RCDNA | G | A | G | C | AG | G | T | C | G | C | C | G | G | G | G | T | C | T | A | C | A | A |
|  | liver RCDNA | G | A | G | C | G | G | T | C | G | C | C | G | G | G | G | T | C | T | A | C | A | A |
|  | liver cccDNA | G | AT | G | C | G | G | T | C | G | C | C | G | G | G | G | T | C | T | A | C | A | A |
| 24 | seum RCDNA | G | T | A | C | G | GA | T | C | G | A | C | G | G | G | G | T | C | T | A | C | A | A |
|  | liver RCDNA | G | A | G | C | G | G | T | C | G | A | C | G | G | G | G | T | C | T | A | C | A | A |
|  | liver cccDNA | G | T | GA | C | G | G | T | C | G | A | C | G | G | G | G | T | C | T | A | C | A | A |
| 25 | seum RCDNA | G | T | GA | C | G | GA | T | C | G | A | C | G | G | G | G | T | C | T | A | C | A | A |
|  | liver RCDNA | G | T | GA | C | G | G | T | C | G | A | C | G | G | G | G | T | C | T | A | C | A | A |
|  | liver cccDNA | G | T | GA | C | G | G | T | C | G | A | C | G | G | G | G | T | C | T | A | C | A | A |
| 26 | seum RCDNA | G | A | G | C | AG | G | T | C | G | C | C | A | G | G | G | T | C | T | A | C | A | A |
|  | liver RCDNA | G | A | G | C | AG | G | T | CA | G | C | C | A | G | G | G | T | C | T | A | C | A | A |
|  | liver cccDNA | G | A | G | C | AG | G | T | C | G | C | C | A | G | G | G | T | C | T | A | C | A | A |
| 27 | seum RCDNA | G | A | G | C | G | G | T | C | G | A | C | G | G | G | G | T | C | T | A | C | A | A |
|  | liver RCDNA | G | A | G | C | G | G | T | C | G | A | C | G | G | G | G | T | C | T | A | C | A | A |
|  | liver cccDNA | G | A | G | C | G | G | T | C | G | A | C | G | G | G | G | T | C | T | A | C | A | A |
| 28 | seum RCDNA | GA | T | GA | C | G | G | T | C | G | A | CA | G | G | G | G | T | C | T | A | C | A | A |
|  | liver RCDNA | GA | T | GA | C | G | G | T | C | G | A | CA | G | G | G | G | T | C | T | A | C | A | A |
|  | liver cccDNA | GA | T | GA | C | G | G | T | C | G | A | CA | G | G | G | G | T | C | T | A | C | A | A |
| 29 | seum RCDNA | G | A | G | C | G | G | T | C | G | A | C | G | G | G | G | T | C | T | A | C | A | A |
|  | liver RCDNA | G | A | G | C | G | G | T | C | G | A | C | G | G | G | G | T | C | T | A | C | A | A |
|  | liver cccDNA | G | A | G | C | G | G | T | C | G | A | C | G | G | G | G | T | C | T | A | C | A | A |
| 30 | seum RCDNA | G | A | G | C | G | G | T | C | G | A | C | G | G | G | G | T | C | T | A | C | GA | A |
|  | liver RCDNA | G | AT | G | C | G | G | T | C | G | A | C | G | G | G | G | T | C | T | A | C | GA | A |
|  | liver cccDNA | G | A | GA | C | G | G | T | C | G | A | C | G | G | G | G | T | C | T | A | C | A | A |
| 31 | seum RCDNA | G | A | G | C | G | G | T | C | G | A | C | G | G | G | G | T | C | T | A | C | GA | A |
|  | liver RCDNA | G | A | G | C | G | G | T | C | G | A | C | G | G | G | G | T | C | T | A | C | A | A |
|  | liver cccDNA | G | A | G | C | G | G | T | C | G | A | C | G | G | G | G | T | C | T | A | C | GA | A |
| 32 | seum RCDNA | G | A | G | C | G | G | T | C | G | A | C | G | G | G | G | T | C | T | A | C | A | A |
|  | liver RCDNA | G | A | G | C | G | G | T | C | G | A | C | G | G | G | G | T | C | T | A | C | A | A |
|  | liver cccDNA | G | A | G | C | G | G | T | C | G | A | C | G | G | G | G | T | C | T | A | C | A | A |
| 33 | seum RCDNA | G | A | G | C | G | G | T | C | G | A | C | G | G | G | G | T | C | T | A | C | A | A |
|  | liver RCDNA | G | A | G | C | G | G | T | C | G | A | C | G | G | G | G | T | C | T | A | C | A | A |
|  | liver cccDNA | G | A | G | C | G | G | T | C | G | A | C | G | G | G | G | T | C | T | A | C | A | A |
| 34 | seum RCDNA | G | T | A | C | G | G | T | C | G | A | C | G | G | G | G | T | C | T | A | C | A | A |
|  | liver RCDNA | G | T | GA | C | G | G | T | C | G | A | C | G | G | G | G | T | C | T | A | C | A | A |
|  | liver cccDNA | G | T | G | C | G | G | T | C | G | A | C | G | G | G | G | T | C | T | A | C | A | A |
| 35 | seum RCDNA | G | A | G | C | G | GA | T | C | G | A | C | G | G | G | G | T | C | TA | A | C | A | A |
|  | liver RCDNA | G | A | G | C | G | GA | T | C | G | A | C | G | G | G | G | T | C | TA | A | C | A | A |
|  | liver cccDNA | G | A | G | C | G | GA | T | C | G | A | C | G | G | G | G | T | C | T | A | C | A | A |
| 36 | seum RCDNA | G | A | G | C | G | G | T | C | G | A | C | G | G | G | G | T | C | T | A | C | A | A |
|  | liver RCDNA | G | A | G | C | G | G | T | C | G | A | C | G | G | G | G | T | C | T | A | C | A | A |
|  | liver cccDNA | G | A | G | C | G | G | T | C | G | A | C | G | G | G | G | T | C | T | A | C | A | A |
| 37 | seum RCDNA | GA | A | G | C | G | GA | T | C | G | A | C | G | G | G | G | T | C | T | A | C | A | A |
|  | liver RCDNA | G | A | G | C | G | G | T | C | G | A | C | G | G | G | G | T | C | T | A | C | A | A |
|  | liver cccDNA | G | A | G | C | G | G | T | C | G | A | C | G | G | G | G | T | C | T | A | C | A | A |
| 38 | seum RCDNA | G | T | A | C | G | G | T | C | G | A | C | G | G | G | G | T | C | T | A | C | A | A |
|  | liver RCDNA | G | T | A | C | G | G | T | C | G | A | C | G | G | G | G | T | C | T | A | C | A | A |
|  | liver cccDNA | G | T | A | C | G | G | T | C | G | A | C | G | G | G | G | T | C | T | A | C | A | A |
| 39 | seum RCDNA | A | T | A | C | G | GA | T | C | G | A | C | G | G | G | G | T | C | TA | A | C | A | A |
|  | liver RCDNA | A | T | A | C | G | GA | T | C | G | A | C | G | G | G | G | T | C | TA | A | C | A | A |
|  | liver cccDNA | A | T | A | C | G | GA | T | C | G | A | C | G | G | G | G | T | C | TA | A | C | A | A |
| 40 | seum RCDNA | G | T | G | C | G | G | T | C | G | A | C | G | G | G | G | T | C | T | A | C | A | A |
|  | liver RCDNA | G | T | G | C | G | G | T | C | G | A | C | G | G | G | G | T | C | T | A | C | A | A |
|  | liver cccDNA | G | AT | G | C | G | G | T | C | G | A | C | G | G | G | G | T | C | T | A | C | A | A |
| 41 | seum RCDNA | G | A | G | C | A | GA | T | C | G | C | C | A | G | G | G | T | C | T | A | C | G | A |
|  | liver RCDNA | G | A | G | CT | A | GA | T | C | G | C | C | A | G | G | G | T | C | T | A | C | G | A |
|  | liver cccDNA | G | A | G | C | A | G | T | C | G | CA | C | A | G | G | G | T | C | T | A | C | G | A |
| 42 | seum RCDNA | G | A | G | C | A | G | T | C | G | A | C | G | G | G | G | T | C | T | A | C | A | A |
|  | liver RCDNA | G | A | G | C | G | G | T | C | G | A | C | G | G | G | G | T | C | T | A | C | A | A |
|  | liver cccDNA | G | A | G | C | G | G | T | C | G | A | C | G | G | G | G | T | C | T | A | C | A | A |
| 43 | seum RCDNA | G | T | CA | C | G | G | T | C | G | A | C | G | G | G | G | A | C | T | A | C | A | A |
|  | liver RCDNA | G | T | CA | C | G | G | T | C | G | A | C | G | G | G | G | A | C | T | A | C | A | A |
|  | liver cccDNA | G | T | CA | C | G | G | T | C | G | A | C | G | G | G | G | A | C | T | A | C | A | A |
| 44 | seum RCDNA | G | A | G | C | G | G | T | C | G | A | C | G | G | G | G | T | C | T | A | C | G | A |
|  | liver RCDNA | G | A | G | C | G | G | T | C | G | A | C | G | G | G | G | T | C | T | A | C | GA | A |
|  | liver cccDNA | G | A | G | C | G | G | T | C | G | A | C | G | G | G | G | T | C | T | A | C | A | A |
| 45 | seum RCDNA | G | T | A | T | A | G | T | C | G | C | C | A | G | G | G | T | C | T | A | C | G | A |
|  | liver RCDNA | G | T | A | T | A | G | T | C | G | C | C | A | G | G | G | T | C | T | A | C | G | A |
|  | liver cccDNA | G | T | A | T | A | G | T | C | G | C | C | A | G | G | G | T | C | T | A | C | G | A |
| 46 | seum RCDNA | G | T | A | C | G | GA | T | C | G | A | C | G | G | G | G | T | C | T | A | C | A | GA |
|  | liver RCDNA | G | AT | GA | C | G | GA | T | C | G | A | C | G | G | G | G | T | C | T | A | C | A | GA |
|  | liver cccDNA | G | T | A | C | G | GA | T | C | G | A | C | G | G | G | G | T | C | T | A | C | A | A |
| 47 | seum RCDNA | G | A | G | C | AG | G | T | C | G | A | C | G | G | G | G | T | C | T | A | C | A | A |
|  | liver RCDNA | G | A | G | C | AG | G | T | C | G | A | C | G | G | G | G | T | C | T | A | C | A | A |
|  | liver cccDNA | G | A | G | C | G | G | T | C | G | A | C | G | G | G | G | T | C | T | A | C | GA | A |
| 48 | seum RCDNA | G | T | A | C | G | G | T | C | G | A | C | G | GA | G | GA | T | C | T | A | C | A | A |
|  | liver RCDNA | G | AT | GA | C | G | G | T | C | G | A | C | G | GA | G | GA | T | C | T | A | C | A | A |
|  | liver cccDNA | G | T | GA | C | G | G | T | C | G | A | C | G | GA | G | G | T | C | T | A | C | A | A |
| 49 | seum RCDNA | G | T | A | C | G | G | T | C | G | A | C | G | G | G | G | T | C | T | A | C | A | GA |
|  | liver RCDNA | G | T | A | C | G | G | T | C | G | A | C | G | G | G | G | T | C | T | A | C | A | GA |
|  | liver cccDNA | G | T | A | C | G | G | T | C | G | A | C | G | G | G | G | T | C | T | A | C | A | A |
| 50 | seum RCDNA | G | A | GA | C | G | G | T | C | G | A | C | G | G | G | G | T | C | T | A | C | A | GA |
|  | liver RCDNA | G | A | G | C | G | G | T | C | G | A | C | G | G | G | G | T | C | T | A | C | A | GA |
|  | liver cccDNA | G | A | GA | CT | AG | GA | T | C | G | A | C | G | G | G | G | T | C | T | A | C | A | GA |
| 51 | seum RCDNA | G | A | A | C | G | G | T | C | G | A | C | G | G | G | G | T | C | T | A | C | A | GA |
|  | liver RCDNA | G | A | A | C | G | G | T | C | G | A | C | G | G | G | G | T | C | T | A | C | A | GA |
|  | liver cccDNA | G | A | G | C | G | G | T | C | G | A | C | G | G | G | G | T | C | T | A | C | A | A |
| 52 | seum RCDNA | G | A | G | CT | GA | G | T | C | G | A | C | G | G | G | G | T | C | T | A | C | A | GA |
|  | liver RCDNA | G | A | G | C | G | G | T | C | G | A | C | G | G | G | G | T | C | T | A | C | A | GA |
|  | liver cccDNA | G | A | G | C | G | G | T | C | G | A | C | G | G | G | G | T | C | T | A | C | A | A |
| 53 | seum RCDNA | A | T | A | T | A | A | T | C | G | C | C | A | G | G | G | T | T | T | A | C | A | A |
|  | liver RCDNA | A | T | A | T | A | A | T | C | G | C | C | A | G | G | G | T | T | T | A | C | A | A |
|  | liver cccDNA | A | T | A | T | A | A | T | C | G | C | C | A | G | G | G | T | T | T | A | C | A | A |
| 54 | seum RCDNA | G | A | G | C | G | GA | T | C | G | A | C | G | G | G | G | T | C | T | A | C | A | A |
|  | liver RCDNA | G | A | G | C | G | GA | T | C | G | A | C | G | G | G | G | T | C | T | A | C | A | A |
|  | liver cccDNA | G | A | G | C | G | G | T | C | G | A | C | G | G | G | G | T | C | T | A | C | A | A |
| 55 | seum RCDNA | A | T | G | C | G | G | T | C | G | A | C | G | G | G | G | T | C | T | CA | C | A | A |
|  | liver RCDNA | A | T | G | C | G | G | T | C | G | A | C | G | G | G | G | T | C | T | A | C | A | A |
|  | liver cccDNA | A | T | G | C | G | G | T | C | G | A | C | G | G | G | G | T | C | T | A | C | A | A |
| 56 | seum RCDNA | G | A | G | CT | G | GA | T | C | G | C | C | A | G | G | G | T | C | T | A | C | A | A |
|  | liver RCDNA | G | A | GA | CT | AG | GA | T | C | G | C | C | A | G | G | G | T | C | T | A | C | A | A |
|  | liver cccDNA | G | A | G | CT | AG | GA | T | C | G | CA | C | A | G | G | G | T | C | T | A | C | A | A |
| 57 | seum RCDNA | G | AT | G | C | G | G | C | T | G | C | C | A | G | G | G | T | C | T | A | C | A | A |
|  | liver RCDNA | G | A | G | C | G | G | C | T | G | C | C | A | G | G | G | T | C | T | A | C | GA | A |
|  | liver cccDNA | G | A | G | C | G | G | C | T | G | C | C | A | G | G | G | T | C | T | A | C | GA | A |
| 58 | seum RCDNA | G | A | G | C | G | G | T | C | G | A | C | G | G | G | G | T | C | T | A | C | A | A |
|  | liver RCDNA | G | A | G | C | G | G | T | C | G | A | C | G | G | G | G | T | C | T | A | C | A | A |
|  | liver cccDNA | G | A | G | C | G | G | T | C | G | A | C | G | G | G | G | T | C | T | A | C | A | A |
| 59 | seum RCDNA | G | A | G | C | G | G | T | C | G | A | T | G | G | G | G | T | C | T | A | C | A | A |
|  | liver RCDNA | G | A | G | C | G | G | T | C | G | A | C | G | G | G | G | T | C | T | A | C | GA | A |
|  | liver cccDNA | G | A | G | C | G | G | T | C | G | A | C | G | G | G | G | T | C | T | A | C | A | A |
| 60 | seum RCDNA | G | A | G | C | AG | A | T | C | G | A | C | G | G | G | G | T | C | T | A | C | A | A |
|  | liver RCDNA | G | A | G | C | AG | A | T | C | G | A | C | G | G | G | G | T | C | T | A | C | A | A |
|  | liver cccDNA | G | A | G | C | G | A | T | C | G | A | C | G | G | G | G | T | C | T | A | C | A | A |
| 61 | seum RCDNA | G | A | G | C | A | GA | T | C | G | A | C | G | GA | G | G | T | C | T | A | C | A | A |
|  | liver RCDNA | G | A | G | C | G | G | T | C | G | A | C | G | G | G | G | T | C | T | A | C | A | A |
|  | liver cccDNA | G | A | G | CT | G | G | T | C | G | A | C | G | G | G | G | T | C | T | A | C | A | A |
| 62 | seum RCDNA | G | A | G | C | G | G | T | C | G | A | C | G | G | G | G | T | C | T | A | C | A | A |
|  | liver RCDNA | G | A | G | C | G | G | T | C | G | A | C | G | G | GT | G | T | C | T | A | C | A | A |
|  | liver cccDNA | G | A | G | C | G | GA | T | C | G | A | C | G | G | G | G | T | C | T | A | C | A | A |
| 63 | seum RCDNA | G | A | G | C | G | GA | T | C | G | A | C | G | G | G | G | T | C | T | A | C | A | A |
|  | liver RCDNA | G | A | G | C | G | GA | T | C | G | A | C | G | G | G | G | T | C | T | A | C | A | A |
|  | liver cccDNA | G | A | G | C | G | GA | T | C | G | A | C | G | G | G | G | T | C | T | A | C | A | A |
| 64 | seum RCDNA | G | A | A | C | G | G | T | C | G | A | C | G | G | G | G | T | C | T | A | C | A | A |
|  | liver RCDNA | G | A | G | C | G | GA | T | C | G | A | C | G | G | G | G | T | C | T | A | C | A | A |
|  | liver cccDNA | G | A | G | C | G | G | T | C | G | A | C | G | G | G | G | T | C | T | A | C | A | A |
| 65 | seum RCDNA | G | T | A | T | G | A | T | C | G | C | C | A | G | G | G | T | C | T | A | C | A | A |
|  | liver RCDNA | G | T | A | T | G | GA | T | C | G | C | C | A | G | G | G | T | C | T | A | C | A | A |
|  | liver cccDNA | G | T | A | T | G | GA | T | C | G | C | C | A | G | G | G | T | C | T | A | C | A | A |
| 66 | seum RCDNA | G | T | A | C | G | G | T | C | G | A | C | G | G | G | G | T | C | T | A | C | A | A |
|  | liver RCDNA | G | TA | A | C | G | G | T | C | G | A | C | G | G | G | G | T | C | T | A | C | A | A |
|  | liver cccDNA | G | A | G | C | G | G | T | C | G | A | C | G | G | G | G | T | C | T | A | C | A | A |
| 67 | seum RCDNA | G | A | G | C | AG | G | T | C | G | A | C | G | G | G | G | T | C | T | A | C | A | A |
|  | liver RCDNA | G | A | G | C | AG | G | T | C | G | A | C | G | G | G | G | T | C | T | A | C | A | A |
|  | liver cccDNA | G | A | G | CT | A | G | T | C | G | A | C | G | G | G | G | T | C | T | A | C | A | A |

Supplementary Table S1 MS results containing all variable sites.
