# Supplementary figures and images for: Differences in sequences between HBV-relaxed circular DNA and covalently closed circular DNA
Source: Emerg Microbes Infect. 2017 Jun 21;6(6):e55–. doi: 10.1038/emi.2017.41 (PMC5520316; doi:10.1038/emi.2017.41)

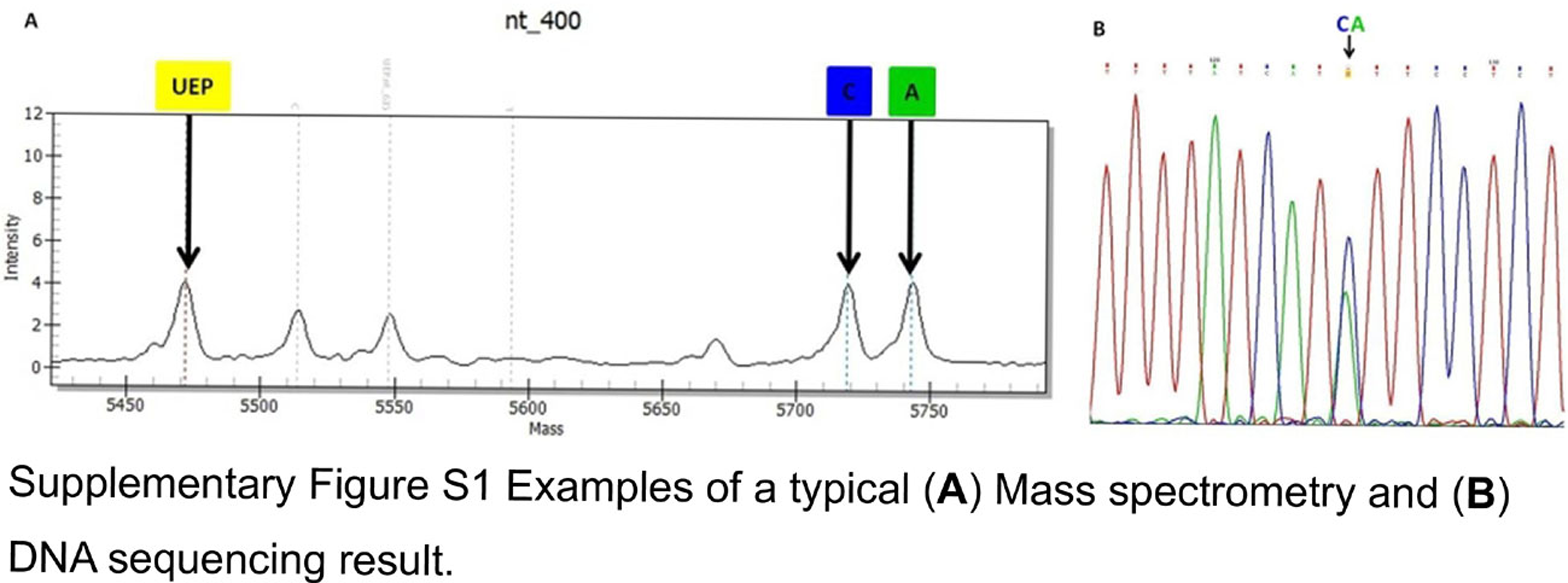

Supplement: Supplementary Figure S1 [file emi201741x2.tif]
